# Potential anti-COVID-19 activity of Egyptian propolis using computational modeling

**DOI:** 10.2217/fvl-2020-0329

**Published:** 2021-02-01

**Authors:** Bassma H Elwakil, Marwa M Shaaban, Adnan A Bekhit, Moustafa Y El-Naggar, Zakia A Olama

**Affiliations:** 1 ^1^Department of Medical laboratory technology, Faculty of Applied Health Sciences Technology, Pharos University in Alexandria, Alexandria, Egypt; 2 ^2^Department of Pharmaceutical Chemistry, Faculty of Pharmacy, Alexandria University, Alexandria 21521, Egypt; 3 ^3^Pharmacy Program, Allied Health Department, College of Health & Sport Sciences, University of Bahrain P.O. Box 32038, Bahrain; 4 ^4^Department of Botany & Microbiology, Faculty of Science, Alexandria University, Alexandria 21568, Egypt

**Keywords:** anti-COVID-19, computational modeling, propolis, remdesivir, umifenovir, lopinavir

## Abstract

**Aim:** To investigate the prospective anti COVID-19 activity of Egyptian propolis. **Material & methods:** Propolis samples were collected from different Egyptian geographical areas and characterized using standardized methods, scanning electron microscope and gas chromatography/mass spectrometry along with computational modeling to predict the anti-COVID-19 activity. **Results & conclusion:** Gas chromatography/mass spectrometry analysis of Menoufia propolis proved the presence of Octatriacontyl pentafluoropropionate (4.2%). Docking analyses declared that Octatriacontyl pentafluoropropionate is well oriented inside the enzyme pockets, in addition to excellent binding manner with the active site of the target macromolecules (RNA-dependent RNA polymerase, Spike protein S1 and main protease) in relation to some broad-spectrum antiviral agents. Menoufia propolis could be a promising candidate in the combat against the pandemic COVID-19.

Recently coronavirus SARS-CoV-2 (COVID-19) has emerged as a new and even fatal disease to humans. It is proved to be originally linked with several pneumonia cases in Wuhan, China. Global clinical cases reached 1,382,658 deaths [1,2]. COVID-19 is genetically related to the beta-coronavirus group, as SARS-coronaviruses, its spike protein has the ability to bind to the human ACE-2 receptor. Coronavirus (CoV) cell infection starts by viral entry, through recognition of ACE-2 and then CoV membrane fuses with the host cell membrane [3]. COVID-19 is a single-stranded RNA virus with a 5′-untranslated region (UTR), nonstructural proteins (nsps) encoded in a replicase complex (orf1ab), gene of a spike protein (S), gene of envelope protein (E), gene of a membrane protein (M), gene of a nucleocapsid protein (N), 3′-UTR, and several unidentified nonstructural open reading frames [1]. Broad-spectrum antiviral agents (BSAAs) are now being considered as potent drug candidates [4]. BSAAs have an advantage of targeting more than two viral families and prevent the viral replicative mechanisms [5]. By spanning Phase II through IV only few existing BSAAs are now being under test in clinical trials. Umifenovir can inhibit the viral entry through membrane fusion inhibition. Umifenovir has been considered in a Phase IV clinical trials combined with different drugs as a potential treatment for pneumonia associated with COVID-19 (ClinicalTrials.gov ID: NCT04255017) [6]. Remdesivir (RNA-dependent RNA polymerase inhibitor) is being considered for mild-to-moderate SARS-CoV-2 in Phase III level clinical trials (ClinicalTrials.gov Identifier: NCT04252664) [7].

Propolis is a well-known natural product and has been used in folk medicine for centuries. It is a resinous material collected from different plant parts by honeybees (*Apis mellifera L*) [8]. Recently propolis has attracted great interest as a potential antimicrobial agent [9]. Propolis composition is primarily affected by the geographical and climatic changes as well as the botanical origin [10]. Propolis' biological activities are correlated with the presence of flavonoids, aromatic acids, diterpenic acids and phenolic compounds [11]. Propolis ethanolic extract has been reported to possess various biological activities, such as antibacterial, antifungal, antiviral, antioxidant, anti-inflammatory, antihepatotoxic and antitumor [7]. Sforcin [12] reported the safety profile of the propolis as a nontoxic compound to humans or mammals with DL50 ranged from 2 to 7.3 g/kg in mice; however for humans DL50 reached 1.4 mg/kg (~70 mg/day).

Novelty impeded in the present work is that propolis samples from different Egyptian geographical areas were collected and comparative chemical composition was conducted through analytical and chromatographic methods with concomitant to the anti-COVID-19 activity.

## Materials & methods

### Propolis samples

Propolis samples were collected from different geographic areas in north Egypt namely: Alexandria (31.21564°N, 29.95527°E), Tanta (30.79611491°N, 30.99517822°E) and Menoufia (30.637468°N, 30.914927°E). The samples were collected and kept in dark sterile glass containers until further use.

### Propolis analysis

#### Scanning electron microscopic examination of propolis samples

Propolis samples were examined using scanning electron microscope JEOL JSM-6390LV (USA). Each sample was oven dried (105˚C/45 min), then coated with metallic gold ions and stored in plastic boxes, which were sealed with parafilm (PARAFILM 1M) to prevent moisture absorption and left at room temperature for 24 h. The samples were then analyzed at different magnifications (Voltage 12 kV, Working Distance 12 mm, Spot size 44, Vacuum Mode HV) [13].

#### Preparation of the propolis extract

Propolis samples were chopped into small pieces and extracted (20% w/v) by maceration with ethanol (99.9%) for 7 days in the dark with continuous stirring followed by sonification for one hour at 70°C [14] then filtered through a Whatman filter paper No. 1 and the extracts were stored in a dark jar at 4°C for further analysis.

#### Chemical analysis

##### Qualitative method

Standard procedures were used to determine the presence or absence of different secondary metabolites of propolis ethanolic extracts such as phenolic compounds, terpenoids, steroids, flavonoids, tannins and glycosides [15].

##### Quantitative method

###### Gas chromatography/mass spectrometry analysis of different propolis samples

About 2 mg of the propolis ethanolic extract was dissolved in 20 μl dry pyridine, then 30 μl N,0-bis(trimethylsilyl)trifluoroacetamide (BSTFA) were added. The mixture was heated at 80°C for 20 min and diluted with 100 μl pyridine then each sample was analyzed using gas chromatography/mass spectrometry (GC/MS). Compounds identification was done using computer search user-generated reference libraries, incorporating mass spectra (Wiley 138 and Nist 98 libraries) [16].

#### Molecular modeling

In the current study, molecular docking was performed to predict the binding affinity of the test compounds toward the target enzymes RNA-dependent RNA polymerase (RdRp), Spike protein S1 and main protease (3CLpro, or 3-chymotrypsin-like protease). This study could describe mode of binding of screened compounds to the active site of target macromolecules and type of interactions of different conformations. The scoring function (S) could compute the binding energy to select orientations with the lowest energy state. It also could be used as indicator of relative strength of interactions with the active site [17].

Molecular Operating Environment (MOE) software was utilized in all the docking experiments, with the x-ray crystal structures of the docking targets; optimized SARS-CoV-2 RdRp (PDB ID: 7BTF), SARS-CoV-2 Spike protein S1 (PDB ID: 6VW1) and SARS-CoV-2 3CLpro (PDB ID: 6Y2G) models were obtained from the Protein Data Bank (PDB) website.

Molecular docking results are often validated using a training set of experimental ligand–protein complexes, and the accuracy of these docking programs is mainly dependent on the used training set [18]. Due to the lack of known ligands, it is important to ensure that the used software is able to replicate the binding mode of a known experimental inhibitor for the studied enzymes. Although neither an effective antiviral drug nor a vaccine against COVID-19 is currently available, several reports have indicated that RdRp inhibitors (remdesivir), influenza glycoproteins inhibitors (umifenovir), in addition to HIV-1 protease inhibitors (lopinavir) have the potential for designing active SARS-CoV-2 inhibitors [19,20]. In the attempt to have reference values (positive control), remdesivir, umifenovir and lopinavir antiviral drugs were considered as comparative standards for the molecular docking.

## Results

### Propolis analysis

Propolis used in the present study was collected from different geographic areas namely: Alexandria, Tanta and Menoufia. Alexandria and Menoufia propolis were dark brown while Tanta propolis was light brown. Propolis classification showed variations according to the botanical origin due to the difference in the dominant pollen grains [21]. Pollen grain analysis was investigated according to Temizer *et al.* [21], which revealed the relatedness of Alexandria propolis to the family Cyperaceae (that was occasionally found in Tanta propolis) while that of Tanta and Menoufia were related to the family Asteraceae.

#### Scanning electron microscopic examination of propolis samples

**Figure 1.**
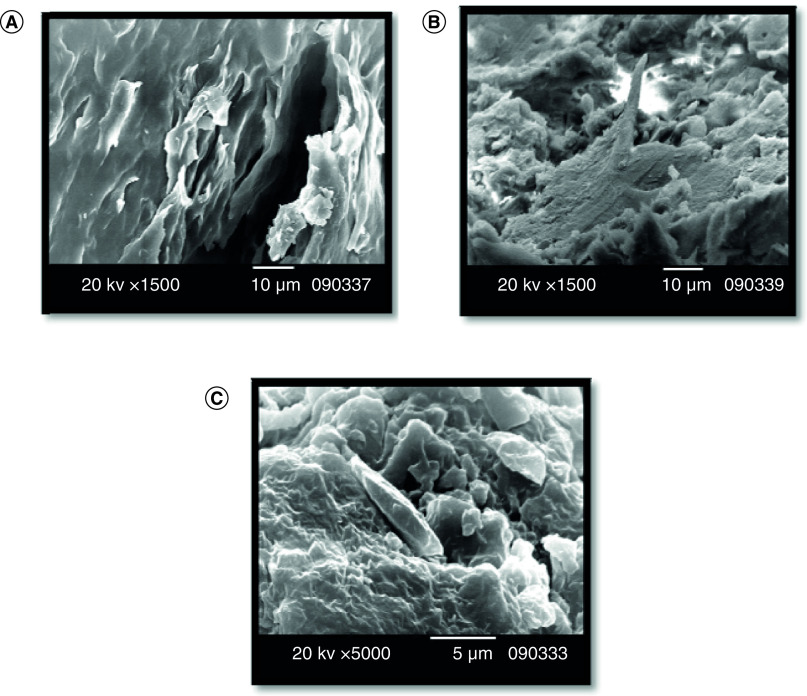
Egyptian propolis analyses and potentail anti COVID-19 effect. Scanning electron micrographs of the Propolis' samples: Alexandria **(A)**, Tanta **(B)** and Menoufia **(C)**.

Scanning electron microscopic analysis of the propolis samples under test (Figure 1A–C) showed 3D structures and morphological features of the propolis samples. It was revealed that no foreign materials were detected and wrinkled surfaces covered with wax layers were observed.

### Chemical analysis of the propolis

#### Qualitative analysis

The qualitative chemical analysis for the tested propolis samples showed variations among the samples that were geographically different. Steroids were not detected in all the tested samples. In the present study a detailed investigation on the composition of the Egyptian propolis collected from specific areas was analyzed (Table 1).

**Table 1.** Qualitative chemical analysis of the propolis samples.

Content	Alexandria	Tanta	Menoufia
Glycosides	+	+	-
Flavonoids	+	+	+
Phenols	-	+	-
Steroids	-	-	-
Tannins	-	+	+
Terpenoids	+	+	+

(+): Present, (-): Absent.

#### Quantitative analysis

##### GC/MS analysis

**Figure 2.**
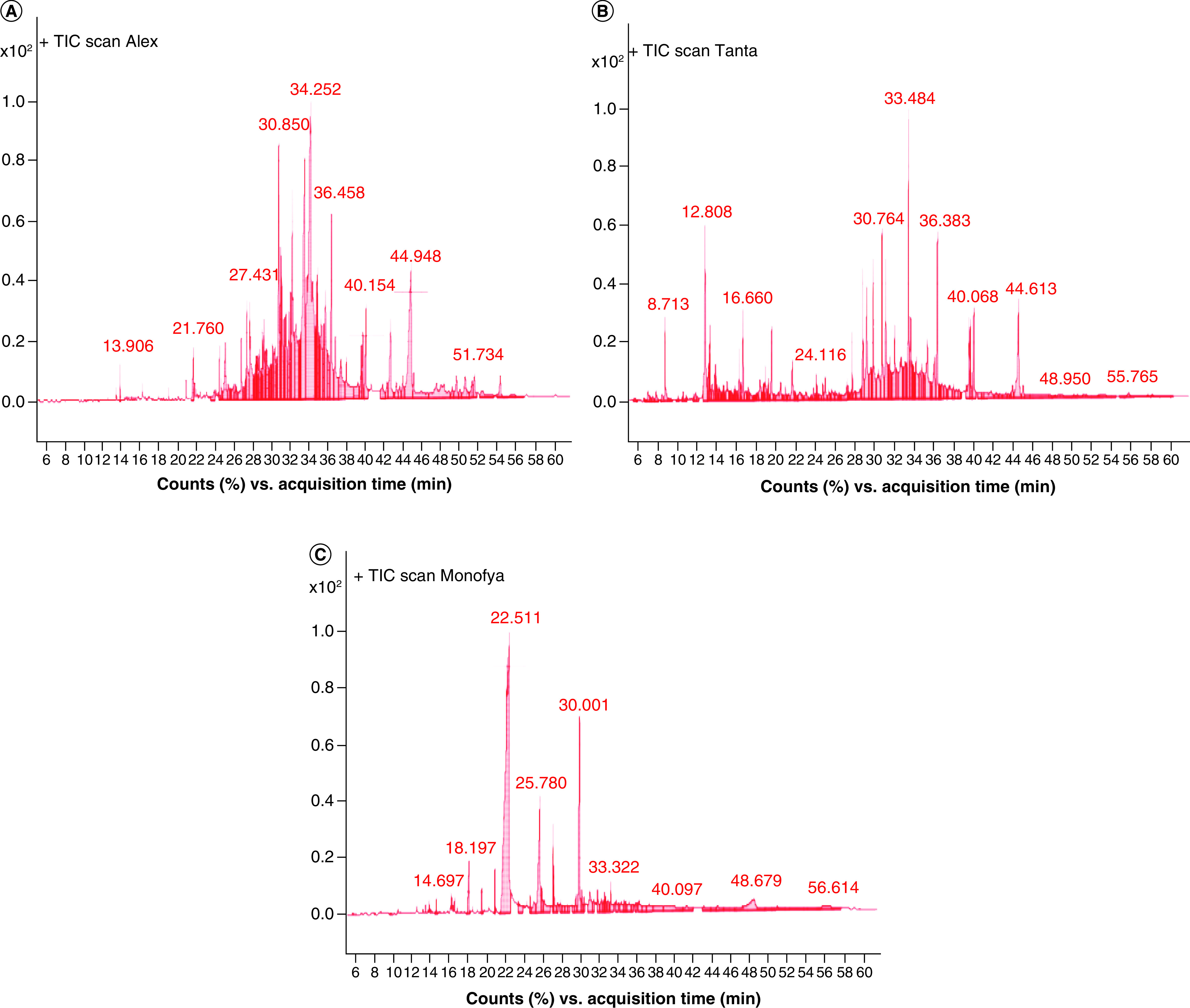
Egyptian propolis analyses and potentail anti COVID-19 effect. Gas chromatography chromatogram of the different propolis samples: Alexandria **(A)**, Tanta **(B)** and Menoufia **(C)**.

On referring the results of the present work to the data base with the corresponding acquisition time (Figures 2A–C), 26 compounds have been identified. It was revealed that the common detected components in the tested extracts were heptacosane, octacosanol and pinocembrin that were in average concentrations except for that of Menoufia and Alexandria propolis, respectively (Table 2). The presence of unusual substances such as sterol precursors and some alcohols may be due to the different geographic areas of propolis in Egypt.

**Table 2.** Gas chromatography/mass spectrometry analysis of the propolis samples under test.

Probable compound	Relative percentage (%)		
	Alexandria	Tanta	Menoufia
**Acids**			
n-Hexadecanoic acid	15.30	-	65.80
trans-13-octadecenoic acid	13.40	-	2.20
Benzoic acid	-	77.50	-
Trans-caffeic acid	-	62.20	-
Tetradecanoic acid	-	-	5.50
**Alkanes**			
Heneicosane	-	5.1	-
Octacosane	-	53	-
Heptacosane	70.60	54.5	-
**Esters**			
Hexadecanoic acid, methyl ester	-	-	1.60
Methyl pentafluoropropionate	-	2.90	-
Pinostrobin chalcone	-	49.90	17.60
Hexadecaneperoxoic acid, 1,1-dimethyl-3-[(1-oxohexadecyl)oxy]propyl ester	-	-	4.20
Oxalic acid, dodecyl 2-phenylethyl ester	-	-	3.40
**Triterpenoids**			
R1-Barrigenol	8.60	-	-
α-Eudesmol	-	26.10	-
β-Eudesmol	-	28.70	-
**Alcohol**			
Octacosanol	34.90	58.80	-
**Flavonois**			
Pinocembrin	-	57.00	17.60
**Not specified**			
Octatriacontyl pentafluoropropionate	-	-	4.2
Bicyclo[2.2.2]octa-2,5-diene, 1,2,3,6-tetramethyl	-	-	1.30
Estra-1,3,5(10)-trien-17-one, 3-hydroxy-2-methoxy	14.60	-	-
4β-Methylandrostane2,3-diol-1,17-dione	27.20	-	-
Pregnan-20-one, 3,17-dihydroxy-, (3β,5β)	11.60	-	-
Octadecane, 3-ethyl-5-(2-ethylbutyl	22.30	-	-
Pregn-5-en-20-one, 11-(acetyloxy)-3,14-dihydroxy-12-(2-hydroxy-3-methyl-1-oxobutoxy)-, (3β,11α,12β,14β)	34.90	-	-
Cyclohexamine, N-n-butyl-1-[2-thionaphthenyl]	25.40	-	-

(-): Not detected.

### Anti-COVID-19 activity using molecular docking

In the current study, molecular docking was performed to predict the binding affinity of the test compounds toward the target enzymes RNA-dependent RNA polymerase, spike protein S1 and main protease (Table 3). Results of the docking studies showed that most compounds possessed promising binding scores, in addition to excellent binding manner with the active site of target macromolecules in comparison to the reference antiviral drugs remdesivir, lopinavir and umifenovir. The binding interactions of test compounds were investigated to study their modes of binding and orientations related to their antiviral activity.

**Table 3.** The binding scores of test compounds toward three protein targets (RNA-dependent RNA polymerase, spike protein S1 and main protease) from 2019-nCoV.

Test compound	Propolis sample source	Binding score (kcal/mol)		
		RNA-dependent RNA polymerase	Spike protein S1	Main protease
Remdesivir		-6.77		
KALETRA^®^ (lopinavir)				-8.18
Arbidol^®^ (umifenovir)			-5.56	
n-Hexadecanoic acid	Alexandria, Menoufia	-5.70	-5.64	-6.28
Benzoic acid	Tanta	-3.91	-4.16	-3.70
Trans-caffeic acid	Tanta	-4.8	-4.76	-4.48
Tetradecanoic acid	Menoufia	-5.34	-5.23	-5.84
Heneicosane	Tanta	-6.19	-5.68	-6.44
Octacosane	Tanta	-6.87	-5.99	-7.39
Heptacosane	Alexandria, Tanta	-6.91	-5.80	-6.95
Hexadecanoic acid, methyl ester	Menoufia	-5.63	-5.27	-6.28
Pinostrobin chalcone	Tanta, Menoufia	-5.59	-5.50	-5.44
Hexadecaneperoxoic acid, 1,1-dimethyl-3-[(1-oxohexadecyl)oxy]propyl ester	Menoufia	-8.04	-6.56	-7.35
Oxalic acid, dodecyl 2-phenylethyl ester	Menoufia	-6.94	-5.95	-6.94
trans-13-octadecenoic acid	Alexandria, Menoufia	-5.97	-5.29	-6.06
Methyl pentafluoropropionate	Tanta	-3.54	-3.55	-3.40
R1-Barrigenol	Alexandria	-5.58	-4.44	-5.81
α-Eudesmol	Tanta	-4.47	-4.57	-4.61
β-Eudesmol	Tanta	-4.96	-4.96	-4.77
Octacosanol	Alexandria, Tanta	-6.96	-6.26	-6.77
Pinocembrin	Tanta, Menoufia	-4.93	-4.78	-5.29
Bicyclo[2.2.2]octa-2,5-diene, 1,2,3,6-tetramethyl	Menoufia	-3.84	-3.93	-4.23
Estra-1,3,5(10)-trien-17-one, 3-hydroxy-2-methoxy	Alexandria	-5.31	-5.26	-6.16
Pregnan-20-one, 3,17-dihydroxy-, (3β,5β)	Alexandria	-5.06	-4.36	-5.53
Octadecane, 3-ethyl-5-(2-ethylbutyl)	Alexandria	-6.11	-5.83	-6.55
4β-Methylandrostane2,3-diol-1,17-dione	Alexandria	-4.72	-4.64	-5.38
Octatriacontyl pentafluoropropionate	Menoufia	-8.20	-6.96	-7.35
Pregn-5-en-20-one, 11-(acetyloxy)-3,14-dihydroxy-12-(2-hydroxy-3-methyl-1-oxobutoxy)-, (3β,11α,12β,14β)	Alexandria	-6.63	-5.03	-6.08
Cyclohexamine, N-n-butyl-1-[2-thionaphthenyl]	Alexandria	-4.90	-5.51	-5.33

#### RNA-dependent RNA polymerase inhibition screening

Considering RNA-dependent RNA polymerase inhibition screening, interestingly, octacosane, heptacosane, hexadecaneperoxoic acid, 1,1-dimethyl-3-[(1-oxohexadecyl) oxy] propyl ester, oxalic acid, dodecyl 2-phenylethyl ester, octatriacontyl pentafluoropropionate and octacosanol possessed remarkable binding scores higher than the reference remdesivir. While, heneicosane, bis(2-ethylhexyl) ester, octadecane, 3-ethyl-5-(2-ethylbutyl) and Pregn-5-en-20-one, 11-(acetyloxy)-3,14-dihydroxy-12-(2-hydroxy-3-methyl-1-oxobutoxy)-, (3β,11α,12β,14β) showed significant binding scores which are quite similar to remdesivir (Table 3).

**Figure 3.**
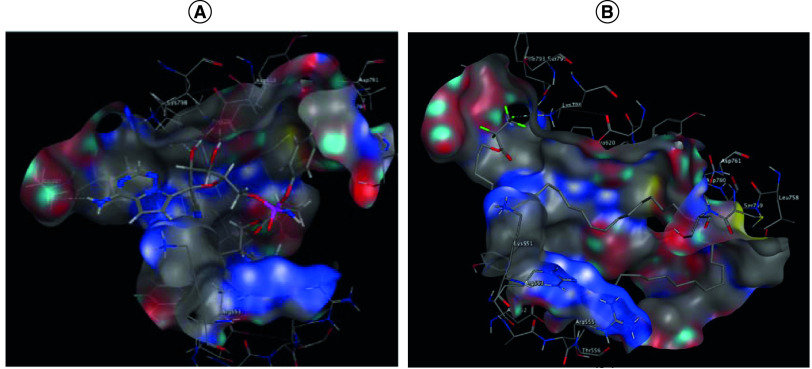
Egyptian propolis analyses and potentail anti COVID-19 effect. Mode of binding of remdesivir **(A)**, and octatriacontyl pentafluoropropionate **(B)** inside RNA-dependent RNA polymerase active site from 2019-nCoV.

The binding site of RdRp was explored computationally, which provides some insights in the discovery of antiviral drugs. The interaction of RdRp with the reference drug remdesivir showed hydrogen bond interaction with Glu167, Asp618, Lys621 and Lys798 (Figure 3A). Docking results for octatriacontyl pentafluoropropionate declared that this compound is well oriented inside the enzyme pockets and showed hydrogen bond interaction with Lys798 (Figure 3B).

#### Spike protein S1 inhibition screening

Results of spike protein inhibition screening indicated that most compounds exhibited significant binding scores similar to those exhibited by the reference drug umifenovir. n-hexadecanoic acid, heneicosane, octacosane, heptacosane, hexadecaneperoxoic acid, 1,1-dimethyl-3-[(1-oxohexadecyl)oxy]propyl ester, oxalic acid, dodecyl 2-phenylethyl ester, bis(2-ethylhexyl) ester, octacosanol, octadecane, octatriacontyl pentafluoropropionate and 3-ethyl-5-(2-ethylbutyl) showed the best scoring results (higher than Umifenovir). While, tetradecanoic acid, hexadecanoic acid, methyl ester, pinostrobin chalcone, trans-13-octadecenoic acid, estra-1,3,5(10)-trien-17-one, 3-hydroxy-2-methoxy, pregn-5-en-20-one, 11-(acetyloxy)-3,14-dihydroxy-12-(2-hydroxy-3-methyl-1-oxobutoxy)-, (3β,11α,12β,14β) and cyclohexamine, N-n-butyl-1-[2-thionaphthenyl] possessed comparable binding scores to umifenovir.

**Figure 4.**
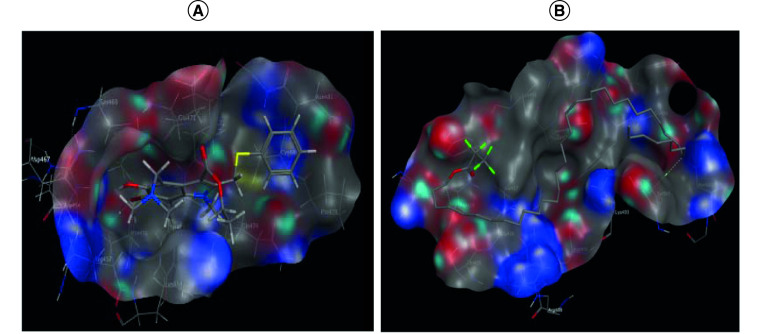
Egyptian propolis analyses and potentail anti COVID-19 effect. Mode of binding of umifenovir **(A)** and octatriacontyl pentafluoropropionate **(B)** inside spike protein S1 active site from 2019-nCoV.

The interaction of spike protein S1 with the reference drug Arbidol^®^ (umifenovir) revealed hydrogen bond interaction with Phe456, in addition to hydrophobic interaction with Lys458 and Asn481 (Figure 4A). Docking results for octatriacontyl pentafluoropropionate showed hydrogen bond interaction with Asp420, in addition to H-arene interaction with Tyr505 (Figure 4B).

#### Main protease enzyme inhibition

Regarding main protease enzyme, octacosane, hexadecaneperoxoic acid, octatriacontyl pentafluoropropionate and 1,1-dimethyl-3-[(1-oxohexadecyl)oxy] propyl ester showed the highest binding scores and good binding profile in comparison to the reference drugs lopinavir.

**Figure 5.**
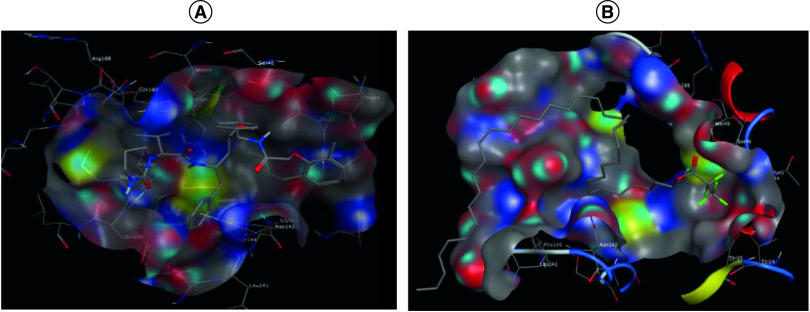
Egyptian propolis analyses and potentail anti COVID-19 effect. Mode of binding of lopinavir **(A)**, and octatriacontyl pentafluoropropionate **(B)** inside main protease 3CLpro active site from 2019-nCoV.

The interaction of 3CLpro with the reference drug KALETRA^®^ (lopinavir) displayed hydrogen bond interaction with Glu166 and hydrophobic interaction with Thr26 and Asn142 (Figure 5A). Docking results for octatriacontyl pentafluoropropionate revealed hydrogen bond interaction with Met49 (Figure 5B).

## Discussion

In the present study, Egyptian propolis samples were analyzed and investigated for their anti-COVID-19 activity using molecular docking. Pollen grain analysis was investigated according to Temizer *et al.* [22] which revealed that the family Cyperaceae was dominant in Alexandria propolis while family Asteraceae was dominant in Tanta and Menoufia propolis samples. Scanning electron microscope analysis of propolis samples revealed that all the propolis samples have a putative gel like morphology. Rahim *et al.* [23] stated that the propolis samples with a homogenized flat topography (with intervals of flaking out and crumpled-like surface topography) may act as building blocks of the hive. Further chemical analysis was applied on the propolis samples, which revealed that the presence of tannins, phenols and steroids varies among the samples which prove that the propolis composition differs according to the correspondence area and the environmental conditions including flora [24]. GC/MS analysis revealed that acids were the main component in the propolis samples. Hegazi *et al.* [24] reported that the characteristic groups of poplar propolis were aliphatic, aromatic acids, aromatic acid esters and flavonoids and it was characterized by the presence of some triterpenoids. Ibrahim *et al.* [25] found that 11 phenolic compounds in propolis samples that were collected from Abees Apiculture Unit, Agricultural Research Farm, University of Alexandria, Alexandria. The phenolic compounds were identified as pinostrobin; izalpinin; tectochrysin; pinocembrin; galangin; chrysin; quercetin-3,3′-di-O-methyl ether; kaempferol-3-O-methyl ether; quercetin-3,7-di-O-methyl ether; isoferulic acid; and galangin-5-O-methyl ether that were not detected in Alexandria propolis (collected from newly reclaimed lands) that was investigated in the present study (Figure 2A & Table 2) which confirmed the relatedness between the chemical constituents, the botanical origin and the seasonal variations.

In the current study, molecular docking was performed to predict the binding affinity [26] of the propolis compounds toward RNA-dependent RNA polymerase, spike protein S1 and main protease (Table 3). Most of the tested fractions showed promising binding scores in relation to the reference antiviral drugs namely: remdesivir, lopinavir and umifenovir. In a trial to investigate the binding interactions of the tested fractions; the modes of binding and orientations were investigated in relation to their antiviral activity. Octatriacontyl pentafluoropropionate showed hydrogen bond interaction with Lys798, Asp420 and Met49, in addition to H-arene interaction with Tyr505 inside RNA-dependent RNA polymerase, spike protein S1 and main protease enzymes pockets, respectively.

## Conclusion

Data from the present study concluded that Egyptian propolis samples proved to have variable chemical composition according to the botanical origin and geographical distribution. Menoufia propolis reported high anti-COVID-19 activity in relation to the tested BSAA and it is recommended as a promising candidate in the combat against the pandemic COVID-19.

**Summary points**

- The present study aimed to investigate the prospective anti-COVID-19 activity of Egyptian propolis.
- Propolis samples were collected from different Egyptian geographic areas.
- Qualitative and quantitative propolis analyses were evaluated. Gas chromatography/mass spectrometry proved the presence of octatriacontyl pentafluoropropionate (4.2%).
- Computational modeling of propolis fractions was evaluated in relation to some known antiviral drugs namely: remdesivir, lopinavir and umifenovir.
- Most of the tested compounds showed a promising binding score.
- Docking analyses declared that octatriacontyl pentafluoropropionate is well oriented inside the enzyme pockets and showed hydrogen bond interaction with Lys798, in addition to excellent binding manner with the active site of the target macromolecules (RNA-dependent RNA polymerase, Spike protein S1 and main protease) in relation to some broad-spectrum antiviral agents.
- Menoufia propolis could be recommended as a promising candidate in the combat against the pandemic COVID-19.
